# Relational similarity-based graph contrastive learning for DTI prediction

**DOI:** 10.1093/bib/bbaf122

**Published:** 2025-03-24

**Authors:** Jilong Bian, Hao Lu, Limin Wei, Yang Li, Guohua Wang

**Affiliations:** College of Computer and Control Engineering, Northeast Forestry University, Harbin 150040, Heilongjiang, China; College of Computer and Control Engineering, Northeast Forestry University, Harbin 150040, Heilongjiang, China; College of Computer and Control Engineering, Northeast Forestry University, Harbin 150040, Heilongjiang, China; College of Computer and Control Engineering, Northeast Forestry University, Harbin 150040, Heilongjiang, China; College of Computer and Control Engineering, Northeast Forestry University, Harbin 150040, Heilongjiang, China

**Keywords:** DTI prediction, graph contrastive learning, relational similarity network, structural features

## Abstract

As part of the drug repurposing process, it is imperative to predict the interactions between drugs and target proteins in an accurate and efficient manner. With the introduction of contrastive learning into drug-target prediction, the accuracy of drug repurposing will be further improved. However, a large part of DTI prediction methods based on deep learning either focus only on the structural features of proteins and drugs extracted using GNN or CNN, or focus only on their relational features extracted using heterogeneous graph neural networks on a DTI heterogeneous graph. Since the structural and relational features of proteins and drugs describe their attribute information from different perspectives, their combination can improve DTI prediction performance. We propose a relational similarity-based graph contrastive learning for DTI prediction (RSGCL-DTI), which combines the structural and relational features of drugs and proteins to enhance the accuracy of DTI predictions. In our proposed method, the inter-protein relational features and inter-drug relational features are extracted from the heterogeneous drug–protein interaction network through graph contrastive learning, respectively. The results demonstrate that combining the relational features obtained by graph contrastive learning with the structural ones extracted by D-MPNN and CNN enhances feature representation ability, thereby improving DTI prediction performance. Our proposed RSGCL-DTI outperforms eight SOTA baseline models on the four benchmark datasets, performs well on the imbalanced dataset, and also shows excellent generalization ability on unseen drug–protein pairs.

## Introduction

When compared to the development of new drugs, drug repurposing often offers lower development costs and shorter development cycles [1]. Therefore, drug repurposing—applying existing medications to the treatment of other diseases—is becoming increasingly attractive [2]. Predicting interactions between drugs and new target proteins (DTIs) is essential for finding new therapeutic applications for existing drugs. Accurate prediction of DTIs is beneficial to better understand the diversity and potential uses of drugs, accelerate drug development and innovation, and provide more options and possibilities for disease treatment [3–5]. DTI prediction is aimed at determining the interactions between proteins and drugs, which enhances our understanding of drug mechanisms in the body and accelerates drug research and development [6]. The rapid advances in computational chemistry and bioinformatics have made it possible to train machine learning models using large amounts of experimental data. Many machine learning approaches have been proposed to efficiently predict DTI and have made significant breakthroughs over the past decade [7–9]. However, with the dramatic increase in experimental data and the increasing complexity of the data distribution, the manually designed features used by traditional machine learning methods cannot meet the complex data distribution, thus limiting their DTI prediction ability. Deep learning-based methods do not need manual feature engineering and selection, instead automatically learn feature representations from raw data. They can fully exploit the advantages of large-scale data, uncovering latent patterns and features within the data to enhance model performance and generalization ability. Consequently, the academic community has proposed many deep learning based DTI prediction approaches [10–13], which typically utilize network models such as CNN, GNN, and RNN to learn their feature representations from a large amount of known drug–target interaction data to predict DTIs. Meanwhile, the feature representations can be used to infer other possible therapeutic purposes for drugs, aiding in drug repurposing.

Generally, deep learning-based DTI prediction can broadly be classified as structural representation learning methods and graph neural network methods. Structural representation learning methods commonly utilize CNN, RNN, and GNN to extract drug features from drug representations, such as SMILES or molecular graphs, as well as protein features from protein representations, such as amino acid sequences. Then, their interaction features are extracted using feature fusion techniques such as concatenation or attention mechanisms to predict DTIs. DeepConv-DTI [14] extracts structural features of proteins and drugs using multi-scale one-dimensional convolution and fully connected layers, respectively. Subsequently, these features are fused through fully connected layers for predicting DTI. DTI-RCNN [15] takes into account the relationship between genes and drugs and combines CNN and LSTM to predict DTI. Since CNN are unable to capture long-range dependencies, Shin et al. [16] proposed the multi-layer bidirectional transformer encoder that builds on the earlier transformer [17] to model dependencies between long-range atoms in drugs. Wang et al. [18] employed protein sequence alignment to produce position specific scoring matrices (PSSMs) of proteins. A stacked autoencoder was then employed to reconstruct the PSSM matrices, thereby obtaining low-dimensional features of proteins. These features were combined with drug molecular fingerprint for DTI prediction. In view of the fact that drug–protein binding actually involves the interaction of the drug with its protein binding sites, Yazdani-Jahromi et al. [19] proposed AttentionSiteDTI, where information about protein binding sites is extracted and modeled as a graph, and the drug is modeled as a graph as well. The topology adaptive graph convolutional networks [20] is then applied to extract drug and protein features. Another category of methods is graph neural network methods following the “guilt-by-association” hypothesis [11, 21]. They use diseases, proteins, drugs, drug side effects, and more to construct heterogeneous networks which are taken as input to heterogeneous graph neural networks to extract node embeddings and edge embeddings for predicting DTIs. The DTINet model proposed by Luo et al. [21] integrates proteins, drugs, and drug side effects into a heterogeneous graph. Then, the low-dimensional feature representations of proteins and drugs are obtained by a compact feature learning algorithm to predict drug–target interactions. Zhou et al. [22] constructed a heterogeneous graph in accordance with DTINet, and then took as inputs the drug SMILES, protein sequences, and the heterogeneous network they form to learn their chemical structure features through sequence region embedding, deep-sampling residual module. Subsequently, in order to take full advantage of multimodal data to obtain a comprehensive embedding representation, they proposed a joint representation based on the heterogeneous graph, which regarded the connected nodes in the heterogeneous graph as the associated nodes, and captured the node correlation by minimizing the distances between the associated nodes. Peng et al. [23] created a heterogeneous graph using drugs, drug side effects, proteins, diseases, and their associations. It then utilized heterogeneous graph convolutional networks to extract features of proteins and drugs for DTI prediction. Li et al. [24] built a drug–target bipartite network using the known DTI data, and computed the sequence similarity between proteins and the chemical structure similarity between drugs as the corresponding node features, respectively. Subsequently, they proposed a personalized propagation graph autoencoder model and a feature embedding model with a bilinear decoder to learn features from bipartite graphs for DTI prediction.

Structural representation learning methods typically take as inputs the amino acid sequences of proteins and drug SMILES to learn the chemical structure features of drugs and proteins. However, this type of methods does not take into account latent inter-drug and inter-protein relational similarities. The predictive accuracy of graph neural network methods relies on the reliability of similarity scores and they generally use the Smith–Waterman algorithm to compute the similarity between protein sequences and the Tanimoto Coefficient (TCC) to calculate the similarity between drugs. However, similar drugs and proteins identified by using these similarity calculation methods do not always guarantee the presence of real interactions between them [25]. Therefore, relying solely on these similarity calculation methods may degrade DTI prediction performance.

With the aim of taking full advantage of the chemical structure information of proteins and drugs, as well as their interrelations, we propose a relational similarity-based graph contrastive learning for DTI prediction( RSGCL-DTI), which uses the structural features of proteins and drugs along with their relational similarity features to predict DTI. Specifically, our proposed RSGCL-DTI first constructs a drug–drug relational graph and a protein–protein relational graph based on known DTI data and employs graph contrastive learning to extract the relational similarity features of drugs and the relational similarity features of proteins, then extracts chemical structure features of drugs and proteins using D-MPNN [26] and CNN, respectively, and finally fuses the relational and structural features of drugs and proteins to predict DTI.

## Method

**Figure 1:**
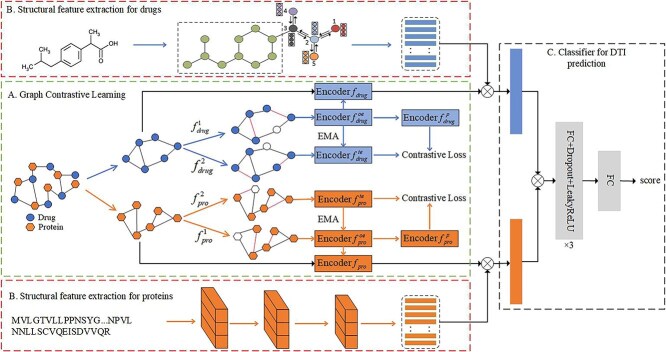
The overall architecture of RSGCL-DTI. (A) The relational features of proteins and drugs are extracted by graph contrastive learning. (B) The structural features of drugs and proteins are extracted by D-MPNN and CNN, respectively. (C) This is the classification module for predicting DTIs.

A novel RSGCL-DTI is proposed in this study. The overall architecture of RSGCL-DTI is depicted in Fig. 1, which is made up of three modules: (i) the relational similarity-based graph contrastive learning module; (ii) the structural feature extraction module; and (iii) the classification module.

In the relational similarity-based graph contrastive learning module, we first utilize known DTI data to construct a heterogeneous graph of drug–target interactions. During construction, if there is an interaction between a drug and a protein, we add an edge between them. After the construction of the heterogeneous graph, it is transformed into a drug–drug relational network and a protein–protein relational network in accordance with the inter-drug relational similarity and the inter-protein relational similarity. Afterwards, graph contrastive learning is adopted to extract drug relational features and protein relational ones. In the structural feature extraction module, the network branch for protein features takes protein sequences as input to extract protein structural features through CNN, while the network branch for drug molecule features takes drug molecule graphs as input to extract drug structural features through D-MPNN. In the classification module, drug and protein relational features are fused with their structural features, then these fused features are concatenated as input to a classifier to predict DTI.

### Relational similarity-based graph contrastive learning module

As a self-supervised deep learning paradigm, contrastive learning has achieved impressive success in image processing field [27–29]. It leverages a large amount of unlabeled data to extract informative and robust features by minimizing discrepancies between different views generated through augmentation of the same data point. In light of this advantage, many researchers have explored contrastive learning on graph-structured data and achieved successful results [30–32]. In this article, a relational similarity-based graph contrastive learning approach is proposed to learn the relational features of proteins and drugs, which, in turn, are used to boost the DTI prediction performance.

### Construction of relational similarity networks

In accordance with the “guilt-by-association” hypothesis [11, 21], drugs that are structurally similar are more likely to have interactions with proteins that are structurally similar. Therefore, we want to use relational data from drugs and proteins to extract their relational features, which will be combined with their structural features to enhance their feature representation. For this purpose, we first derive a relational similarity network among drugs and a relational similarity network among proteins from a heterogeneous graph of drug–target interactions. Then, graph contrastive learning is utilized on the homogeneous graphs to extract relational features for drugs and proteins. Specifically, we first construct a heterogeneous graph $G_{dp}=(V_{d},V_{p},E)$ of drug–target interactions using known DTI data, where $V_{d}$ and $V_{p}$ denote a drug set and a protein set, respectively, $E\in \{0,1\}^{|V_{d}|\times |V_{p}|}$ denotes the drug–protein relational matrix, and $|V_{p}|$ and $|V_{d}|$ denote the number of proteins and drugs, respectively. Each column in $E$ represents the interactions between one protein and all of the drugs, and each row represents the interactions between one drug and all of the proteins. These interactions are used as feature representations of proteins and drugs, and then cosine similarities between drugs and between proteins are computed to construct two relational similarity networks: one for drugs and the other for proteins. To be specific, in constructing relational similarity network for drugs, the feature encodings for drugs $i$ and $j$ are represented by the $i$th and $j$th rows of the $E$, respectively. The features of drugs $i$ and $j$ are denoted as $g_{i}\in{\mathbb{R}^{|V_{p}|}}$ and $g_{j}\in{\mathbb{R}^{|V_{p}|}}$. Their cosine similarity is calculated as follows:


(1) \begin{align*} Sim_{ij}=\frac{g_{i} \cdot g_{j}}{\sqrt{(g_{i})^{2}}\cdot \sqrt{(g_{j})^{2}}}\end{align*}

If the cosine similarity $Sim_{ij}$ between drugs $i$ and $j$ exceeds the drug similarity threshold $\tau _{d}$ that we set, then they are considered to be similar, thereby adding an edge from drug $i$ to drug $j$ in a drug–drug relational similarity network. The selection of similarity thresholds for $\tau _{d}$ is related to the sparsity of the networks. To prevent the relationship similarity network from becoming too sparse to effectively represent the similarity relationships, we choose 0.3 as the similarity threshold in our experiments. By computing pairwise similarities between drugs, we obtain the adjacency matrix $A_{d}\in{\{0,1\}^{|V_{d}|\times |V_{d}|}}$. Next, a random matrix $X_{d}\in{\mathbb{R}^{|V_{d}|\times 167}}$ is generated as the initial node feature matrix, resulting in the drug–drug relational similarity network $G_{d}=(A_{d},X_{d})$. In the same way, we obtain the protein–protein relational similarity network $G_{p}=(A_{p},X_{p})$, where $A_{p}\in \mathbb{R}^{|V_{p}|\times |V_{p}|}$ and $X_{p}\in \mathbb{R}^{|V_{p}|\times 256}$. Note: the initial feature dimension of the drug nodes is 167, which is determined based on the MACCS fingerprints of size 167-bit generated by RDKit, while the initial feature dimension of the protein nodes is 256, which is determined experimentally.

### Relational feature extraction based on contrastive learning

Inspired by graph contrastive learning [31, 33], we mine the implicit relational features from the relational similarity networks for drugs and proteins through graph contrastive learning to boost DTI prediction accuracy. The module architecture of the contrastive learning is displayed in Fig. 1A, which is composed of two graph contrastive learning module: one module for drugs and the other module for proteins. Two augmentation functions, $f_{drug}^{1}$ and $f_{drug}^{2}$, are applied to augment the graph structure by randomly removing edges and randomly setting node features to zero vectors within the drug–drug relational similarity network $G_{d}$, resulting in two contrastive views $G_{d}^{1}=(A_{d}^{1},X_{d}^{1})$ and $G_{d}^{2}=(A_{d}^{2},X_{d}^{2})$. Then, two graph encoders $f_{drug}^{oe}$ and $f_{drug}^{te}$ with identical structures are used to encode $G_{d}^{1}$ and $G_{d}^{2}$, respectively, to obtain two view representations $H_{drug}^{oe}$ and $H_{drug}^{te}$:


(2) \begin{align*} H_{drug}^{oe}=f_{drug}^{oe}\left(f_{drug}^{1}(G_{d})\right) \end{align*}

(3) \begin{align*} H_{drug}^{te}=f_{drug}^{te}\left(f_{drug}^{2}(G_{d})\right) \end{align*}

Next, $H_{drug}^{oe}$ is fed into the predictor $f_{drug}^{p}$ for linear transformation:


(4) \begin{align*} Z_{drug}^{oe}=f_{drug}^{p}\left(H_{drug}^{oe}\right)\end{align*}

Finally, the corresponding nodes in the view representations $H_{drug}^{te}$ and $Z_{drug}^{oe}$ are considered as a pair of positive samples, and the parameters in the contrastive model are updated by minimizing the difference between them.


(5) \begin{align*} L=-\frac{2}{N}\sum_{i = 0}^{N-1}\frac{{Z_{drug}^{oe}}_{i}\cdot{H_{drug}^{te^{T}}}_{i}}{\left\lVert{Z_{drug}^{oe}}_{i}\right\rVert \cdot \left\lVert{H_{drug}^{te}}_{i}\right\rVert }\end{align*}

In order to prevent the contrastive model from converging to trivial solutions, the graph encoder $f_{drug}^{oe}$ utilizes the Adam optimization algorithm for parameter update, while the graph encoder $f_{drug}^{te}$ employs the exponential moving average method for parameter update:


(6) \begin{align*} \xi\leftarrow\tau\xi+(1-\tau)\theta\end{align*}

where $\xi $ is the parameters of $f_{drug}^{te}$, $\theta $ is the parameters of $f_{drug}^{oe}$, and $\tau \in (0,1)$ denotes decay ratio.

After the training of the contrastive learning model, the graph encoder $f_{drug}^{oe}$ is applied to the original drug–drug relational similarity network $G_{d}$ to obtain the drug relational feature matrix $Z_{drug}^{r}\in \mathbb{R}^{|V_{d}|\times 300} $, where each row represents the relational feature $X_{drug}^{r}\in \mathbb{R}^{300}$ of a drug. In the same way, the protein relational feature matrix $Z_{protein}^{r}\in \mathbb{R}^{|V_{p}|\times 300} $ is calculated from the protein–protein relational similarity network.

### Structural feature extraction module

Amino acids are the primary structural units that constitute proteins. The amino acid sequence comprising a protein specifies the protein’s structure and this structure further regulates its function and activity, showing that the sequence of amino acids is critical descriptive information within a protein. For this reason, the sequences of amino acids are chosen as inputs to our model, and CNNs are utilized to extract protein structural features. As a rule, drug activities and properties are related to its molecular structure and topology. Furthermore, a molecular graph can serve as an effective means of representing the structure and topology of a drug. Hence, we choose a molecular graph to represent a drug and utilize D-MPNN to extract its structural features.

### Structural feature extraction module for drugs

In this module, drugs are first converted into molecular graphs by RDKit toolkit. The initial features of nodes in the molecular graph include eight atomic properties: atomic number, bond count, formal charge, chirality, hydrogen count, hybridization, aromaticity, and atomic mass. Additionally, the initial features of edges consist of four bond properties: bond type, conjugation, ring membership, and stereochemistry. Finally, D-MPNN is used to compute the hidden features of all nodes, and the graph-level feature $X_{drug}^{s}\in \mathbb{R}^{300}$ is obtained by pooling the hidden features of all nodes in the readout phase.

### Structural feature extraction module for proteins

In this module, the Tasks Assessing Protein Embeddings tokenizer [34] is adopted to encode the amino acid sequence of a protein into its digital representation, which is then padded with zeros to get a sequence of tokens $x_{token}\in \mathbb{R}^{L_{s}}$ of the fixed length. The pseudo code is shown in Algorithm 1. Then, the sequence of tokens $x_{token}$ is fed into an embedding layer to obtain an embedding matrix. Finally, CNN and MaxPool are applied to extract the protein sequence feature $X_{protien}^{s}\in \mathbb{R}^{300}$:




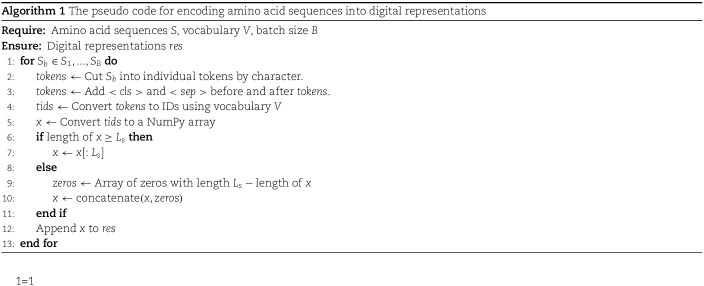




(7) \begin{align*} X_{protein}^{s}=MP(CNN(Emb(x_{token})))\end{align*}

where $MP(\cdot )$ denotes the MaxPool layer, $CNN(\cdot )$ denotes the CNN block, and $Emb(\cdot )$ denotes the embedding layer.

### Classification module

Our proposed model first obtains the relational and structural features of proteins and drugs through graph contrastive learning module and structural feature extraction module, respectively. Subsequently, these relational and structural features are combined through concatenation, respectively:


(8) \begin{align*}& \begin{aligned} X_{drug} &= \text{concat}\left(X_{drug}^{r}, X_{drug}^{s}\right) \\ X_{protein} &= \text{concat}\left(X_{protein}^{r}, X_{protein}^{s}\right) \end{aligned}\end{align*}

where $X_{drug}\in \mathbb{R}^{600}$ and $X_{protein}\in \mathbb{R}^{600}$ are combined drug feature and combined protein feature, respectively. Finally, the combined features $X_{drug}$ and $X_{protein}$ are taken as inputs to the classification module for DTI prediction. Following previous research [14, 19, 35], we take MLP as the classification network and binary cross entropy loss as the loss function. In the MLP, we employ three fully connected layers, each followed by a dropout layer and a LeakyReLU activation function. Next, the output of the MLP is passed through an additional fully connected layer to produce predictions.

## Results

### Dataset and evaluation metrics

To compare RSGCL-DTI with existing DTI prediction methods, we utilized DrugBank [35], BioSNAP [36], Human [37], and C.elegans [37] to assess the performance of all models. The details of these datasets are listed on Table 1. All experiments were conducted using 10-fold cross-validation. The test set was obtained by randomly selecting 10% of the data from the dataset. The remaining 90% of the data were divided into 10 subsets. In each fold, one subset was used as the validation set, while the remaining nine subsets were used as the training set. Positive examples were constructed from known drug–protein interaction pairs in the dataset. The negative examples selection process involved randomly selecting a drug and then randomly choosing a protein from the set of proteins that do not interact with the selected drug to form a negative pair. Then the uniqueness of the generated pair is verified against the existing negative examples. Only non-redundant pairs were incorporated into the final negative example set. On the balanced datasets, ACC, AUC, recall, precision, and AUPR are used to evaluate RSGCL-DTI and baselines, while on the imbalanced datasets, AUPR and AUC are used to assess RSGCL-DTI and baselines.

**Table 1:** The detailed description of benchmark datasets

Dataset	Protein	Drug	Negative	Positive
Human	2001	2726	3364	3364
C.elegans	1876	2726	3894	3893
DrugBank	2888	6459	14442	11885
BioSNAP	2181	4509	13834	13834

### Comparison experiments

To comprehensively assess the predictive capability of our presented RSGCL-DTI model, we selected MHSADTI [38], HyperAttentionDTI [35], GIFDTI [39], MCANet [40], BINDTI [41], MCL-DTI [42], CoaDTI [43], and DRGCL [44] as baselines. In order to guarantee that the evaluation is both scientific and effective, we established the same experimental environment to make a comparison between the baselines mentioned above and the RSGCL-DTI and reported the average metric values under 10-fold CV for the dataset DrugBank, C.elegans, Human, and BioSNAP. When presenting the comparative results, the best metric values are highlighted in bold, while the second-best metric values are underlined.

**Figure 2:**
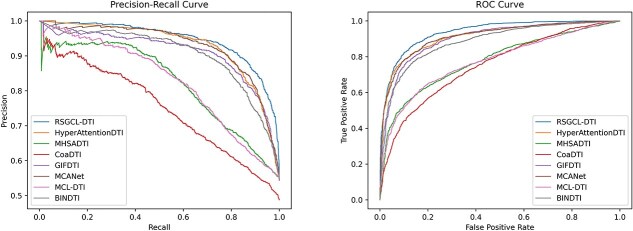
PR and ROC curves of RSHCL-DTI and baselines on DrugBank.

The comparative results on DrugBank are provided in Table 2. Based on the results shown in the table, we can observe that RSGCL-DTI achieved the highest prediction performance in AUC, ACC, precision, and AUPR. RSGCL-DTI outperforms the second-ranked MCANet by 1.43, 2.13, and 2.02% on the AUC, ACC, and AUPR metrics, respectively, and achieves an improvement in precision of 0.87% over BINDTI, which is the baseline model with the highest precision. However, it is ranked second in recall, 0.7% lower than the top-ranking GIFDTI. To provide a more intuitive comparison of all models, we plotted ROC and PR curves for each model using the DrugBank dataset, as shown in Fig. 2.

**Table 2:** The comparative results of RSGCL-DTI and baseline models for DrugBank

Model	AUC(std)	ACC(std)	Recall(std)	Precision(std)	AUPR(std)
RSGCL-DTI	**0.9315**(0.0065)	**0.8658**(0.0142)	0.8912(0.0334)	**0.8683**(0.0122)	**0.9528**(0.0047)
GIFDTI	0.9115(0.0049)	0.8265(0.0085)	**0.8982**(0.0085)	0.8091(0.0106)	0.9201(0.0057)
HyperAttentionDTI	0.9105(0.0033)	0.8272(0.0053)	0.8802(0.0059)	0.8160(0.0084)	0.9289(0.0032)
MHSADTI	0.8695(0.0089)	0.7912(0.0115)	0.8381(0.0254)	0.7967(0.0243)	0.8622(0.1000)
MCANet	0.9172(0.0046)	0.8445(0.0059)	0.8751(0.0147)	0.8487(0.0061)	0.9326(0.0048)
BINDTI	0.8852(0.0056)	0.8148(0.0026)	0.7921(0.0060)	0.8596(0.0019)	0.9088(0.0079)
MCL-DTI	0.8514(0.0036)	0.7834(0.0023)	0.8169(0.0057)	0.7945(0.0041)	0.7743(0.0090)
CoaDTI	0.7849(0.1290)	0.7126(0.0106)	0.5943(0.0824)	0.7356(0.0580)	0.6793(0.0077)

**Figure 3:**
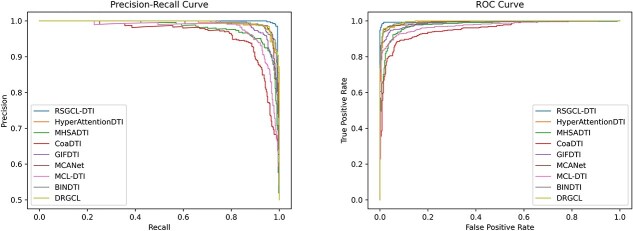
PR and ROC curves of RSHCL-DTI and baselines on C.elegans.

The performance of RSGCL-DTI and baselines for C.elegans is displayed in Table 3, from which we can observe that RSGCL-DTI achieves the best performance on the ACC, recall, precision, and AUPR, outperforming the top-ranked baseline by 1.91, 1.49, 1.54, and 0.18%, respectively. However, it was 0.05% lower in AUC than DRGCL, which is the baseline model with the highest AUC. The ROC and PR curves are provided in Fig. 3.

**Table 3:** The comparative results of RSGCL-DTI and baseline models for C.elegans

Model	AUC(std)	ACC(std)	Recall(std)	Precision(std)	AUPR(std)
RSGCL-DTI	0.9946(0.0008)	**0.9871**(0.0026)	**0.9826**(0.0056)	**0.9924**(0.0063)	**0.9969**(0.0004)
GIFDTI	0.9880(0.0056)	0.9422(0.0135)	0.9529(0.0175)	0.9366(0.0163)	0.9902(0.0049)
HyperAttentionDTI	0.9934(0.0011)	0.9551(0.0088)	0.9677(0.0095)	0.9466(0.0133)	0.9939(0.0011)
MHSADTI	0.9776(0.0062)	0.9242(0.0046)	0.9652(0.0072)	0.9002(0.0063)	0.9808(0.0057)
MCANet	0.9920(0.0018)	0.9661(0.0048)	0.9666(0.0090)	0.9666(0.0054)	0.9925(0.0014)
BINDTI	0.9907(0.0034)	0.9602(0.0018)	0.9628(0.0218)	0.9604(0.0165)	0.9932(0.0019)
MCL-DTI	0.9706(0.0030)	0.9072(0.0063)	0.8486(0.0161)	0.9744(0.0112)	0.9769(0.0031)
CoaDTI	0.9527(0.0016)	0.8921(0.0070)	0.8537(0.0114)	0.9434(0.0063)	0.9629(0.0033)
DRGCL	**0.9951**(0.0016)	0.9680(0.0271)	0.9586(0.0021)	0.9770(0.0421)	0.9951(0.0016)

**Figure 4:**
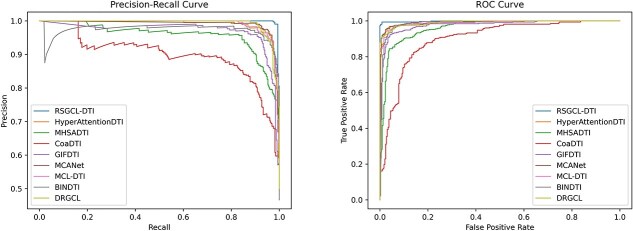
PR and ROC curves of RSHCL-DTI and baselines on Human.

On the Human dataset, RSGCL-DTI has an improvement of 0.26, 1.31, 1.00, and 0.23% in AUC, ACC, recall, and AUPR over the baselines with the highest metric, respectively, but is 0.05% lower in precision than DRGCL, which is a baseline with the highest precision. The experimental results are shown in Table 4. The ROC and PR curves are provided in Fig. 4.

**Table 4:** The comparative results of RSGCL-DTI and baseline models for Human

Model	AUC(std)	ACC(std)	Recall(std)	Precision(std)	AUPR(std)
RSGCL-DTI	**0.9963**(0.0007)	**0.9703**(0.0026)	**0.9715**(0.0043)	0.9677(0.0033)	**0.9925**(0.0006)
GIFDTI	0.9715(0.0051)	0.9140(0.0134)	0.9197(0.0161)	0.9022(0.0342)	0.9807(0.0069)
HyperAttentionDTI	0.9805(0.0026)	0.9348(0.0053)	0.9586(0.0052)	0.9226(0.0065)	0.9516(0.0038)
MHSADTI	0.9693(0.0012)	0.9117(0.0056)	0.9311(0.0142)	0.8992(0.0155)	0.9225(0.0054)
MCANet	0.9937(0.0020)	0.9572(0.0031)	0.9615(0.0098)	0.9519(0.0095)	0.9902(0.0023)
BINDTI	0.9863(0.0028)	0.9535(0.0039)	0.9615(0.0013)	0.9404(0.0059)	0.9719(0.0024)
MCL-DTI	0.9850(0.0027)	0.9459(0.0053)	0.9482(0.0052)	0.9452(0.0065)	0.9516(0.0038)
CoaDTI	0.9700(0.0077)	0.9154(0.0070)	0.9395(0.0255)	0.9061(0.0154)	0.9131(0.0067)
DRGCL	0.9876(0.0031)	0.9476(0.0101)	0.9232(0.0198)	**0.9682**(0.0226)	0.9899(0.0024)

**Figure 5:**
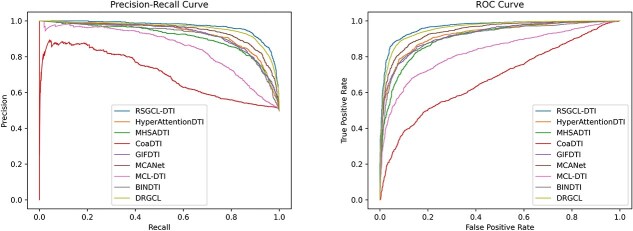
PR and ROC curves of RSHCL-DTI and baselines on BioSNAP.

Table 5 displays the experimental results for the BioSNAP dataset. RSGCL-DTI outperforms the best-performing baseline, DRGCL by 0.04, 0.62, and 0.15% on the AUC, recall, and AUPR, respectively, but is 0.18 and 0.8% lower than DRGCL on the ACC and precision. Figure 5 shows PR and ROC curves of RSGCL-DTI and baselines on BioSNAP. To further assess RSGCL-DTI’s predictive performance, we utilized the water tool from the EMBOSS suite to conduct local sequence alignments of protein sequences from the BioSNAP dataset, thereby obtaining sequence identities. Subsequently, we employed agglomerative clustering to derive 10 clusters, which served as 10 folds to ensure greater independence of data across the folds. The results are shown in Table 6. The experimental results show that the standard deviations of the AUC and AUPR for all models have increased. Moreover, the performance of structure-based DTI prediction methods such as GIFDTI, HyperAttentionDTI, MHSADTI, and MCANet has decreased significantly, while the performance of network-based methods like DRGCL and RSGCL-DTI has decreased only slightly. We believe that this is because when the sequence similarity between the proteins in the test set and those in the training set is low, there may be significant differences in their three-dimensional structures. As a result, structure-based models find it difficult to learn effective patterns from the training set to accurately predict drug–protein interactions in the test set, leading to a substantial decline in performance. In contrast, network-based methods do not rely entirely on the structural information of proteins and drugs. Instead, they utilized the topological structure of the network and the relationships between nodes to identify potential interactions. Therefore, their performance decline is relatively small.

**Table 5:** The comparative results of RSGCL-DTI and baseline models for BioSNAP

Model	AUC(std)	ACC(std)	Recall(std)	Precision(std)	AUPR(std)
RSGCL-DTI	**0.9605**(0.0025)	0.8899(0.0041)	**0.8889**(0.0097)	0.8947(0.0111)	**0.9572**(0.0046)
GIFDTI	0.9179(0.0060)	0.8412(0.0106)	0.8671(0.0126)	0.8265(0.0142)	0.8412(0.0051)
HyperAttentionDTI	0.9228(0.0051)	0.8475(0.0080)	0.8679(0.0116)	0.8357(0.0093)	0.9277(0.0054)
MHSADTI	0.9078(0.0071)	0.8344(0.0099)	0.8410(0.0205)	0.8331(0.0139)	0.8674(0.0067)
MCANet	0.9354(0.0026)	0.8630(0.0068)	0.8467(0.0101)	0.8851(0.0070)	0.9387(0.0041)
BINDTI	0.9118(0.0024)	0.8413(0.0021)	0.8241(0.0060)	0.8652(0.0044)	0.9117(0.0049)
MCL-DTI	0.8809(0.0056)	0.8095(0.0066)	0.8224(0.0370)	0.8043(0.0249)	0.7780(0.0057)
CoaDTI	0.7185(0.0085)	0.6847(0.0118)	0.5814(0.0608)	0.7357(0.0330)	0.7391(0.0066)
DRGCL	0.9601(0.0186)	**0.8917**(0.0228)	0.8827(0.0601)	**0.9027**(0.0447)	0.9557(0.0199)

**Table 6:** The experimental results of agglomerative clustering on the BioSNAP dataset

Model	AUC(std)	AUPR(std)
RSGCL-DTI	0.9128(0.0064)	0.8903(0.0106)
GIFDTI	0.8366(0.0124)	0.8184(0.0151)
HyperAttentionDTI	0.8202(0.0125)	0.8153(0.0172)
MHSADTI	0.8100(0.0499)	0.8001(0.0221)
MCANet	0.8350(0.0111)	0.8306(0.0077)
BINDTI	0.6771(0.0410)	0.6988(0.0554)
MCL-DTI	0.7168(0.0239)	0.7483(0.0524)
CoaDTI	0.7071(0.0631)	0.7258(0.0767)
DRGCL	0.9096(0.0192)	0.8821(0.0213)

**Figure 6:**
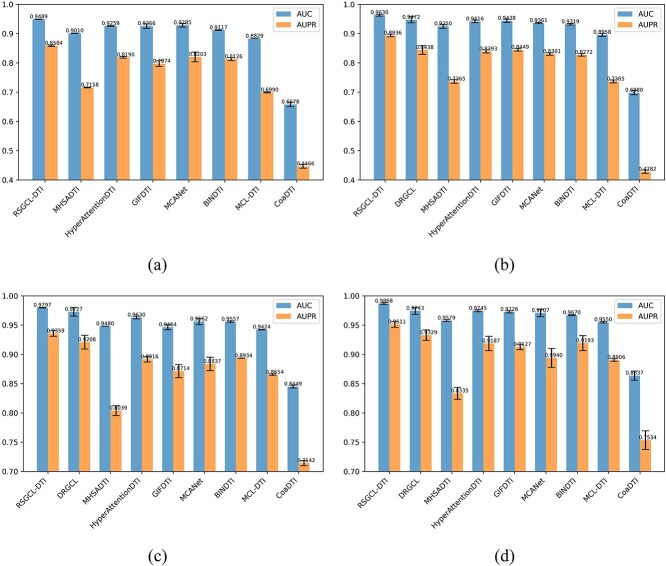
The experimental results on the imbalanced datasets: (a) the results on the imbalanced DrugBank. (b) The results on the imbalanced BioSNAP. (c) The results on the imbalanced Human. (d) The results on the imbalanced C.elegans.

To evaluate the predictive capacity of the RSGCL-DTI model on imbalanced datasets, we constructed four imbalanced datasets based on the DrugBank, BioSNAP, Human, and C.elegans datasets by repeated sampling, with a positive-to-negative example ratio of 1:5. Then we evaluated the performance of RSGCL-DTI and baselines on these imbalanced datasets using AUPR and AUC metrics. We can observe from Fig. 6 that RSGCL-DTI outperforms all baselines, showcasing that RSGCL-DTI also achieved excellent performance on the imbalanced datasets.

**Figure 7:**
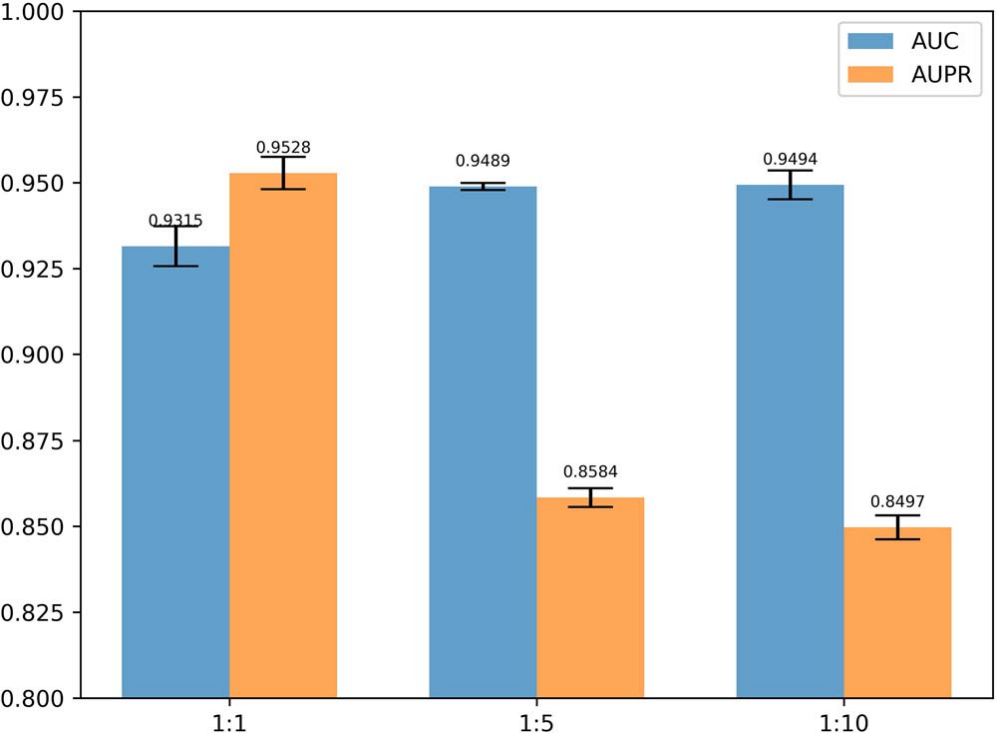
The experimental results of positive and negative examples with different proportions in DrugBank.

In order to explore the changes in the performance of the RSGCL-DTI model when a positive-to-negative ratio in the dataset is gradually increased, we conducted experiments with the DrugBank dataset with a positive-to-negative ratio of 1:1, 1:5, and 1:10. The results of the experiments are shown in Fig. 7. The experimental results show that the model’s AUPR decreases with increasing degrees of dataset imbalance. When the positive-to-negative ratio of the dataset increases from 1:5 to 1:10, the model’s AUPR only decreases by 0.87%, demonstrating that our model has good resistance to data imbalance.

### Ablation experiment

Numerous studies indicate that the interactions between drugs and proteins are influenced by their chemical structures, while latent inter-drug and inter-protein relationships also affect drug–protein interactions. For this reason, we propose to combine the relational features of proteins and drugs with their structural features to improve predictive performance. In order to explore the impact of relational and structural features on predictive performance, we designed two variant models RSGCL-DTI-RF and RSGCL-DTI-SF, where RSGCL-DTI-RF considered only the effects of relational features on DTI prediction accuracy, while RSGCL-DTI-SF considered only the effects of structural features on DTI prediction accuracy. Specifically, RSGCL-DTI-RF only adopts graph contrastive learning to learn relational features for DTI prediction, while RSGCL-DTI-SF only uses CNN and D-MPNN to extract structural features of proteins and drugs for DTI prediction. In addition, to ensure that the experimental comparisons are performed in a fair and reasonable manner, we determined the optimal hyperparameters for the RSGCL-DTI, RSGCL-DTI-RF, and RSGCL-DTI-SF models according to several experiments to ensure the best performance of the models. We performed experiments on the DrugBank dataset to determine the hyperparameters of RSGCL-DTI-RF and RSGCL-DTI-RF, and some of the experimental results are shown in Table 7. We tested the models RSGCL-DTI-RF and GCL-DTI-SF on the DrugBank dataset under 10-fold cv. The results of the ablation experiments for RSGCL-DTI are shown in Table 8.

**Table 7:** The experimental results on determining the hyperparameters of the variant models

Model	Hyperparameter	AUC(std)	AUPR(std)
RSGCL-DTI-SF	One layer of CNN	0.8937(0.0118)	0.9188(0.0085)
	Two layers of CNN	0.9197(0.0026)	0.9192(0.0033)
	Three layers of CNN	0.9071(0.0051)	0.9270(0.0050)
	One layer of D-MPNN	0.8985(0.0090)	0.9175(0.0099)
	Two layers of D-MPNN	0.9197(0.0026)	0.9192(0.0033)
	Three layers of D-MPNN	0.8902(0.0189)	0.9165(0.0126)
RSGCL-DTI-RF	One layer of GCN	0.9164(0.0031)	0.9210(0.0038)
	Two layers of GCN	0.9249(0.0025)	0.9255(0.0045)
	Three layers of GCN	0.9153(0.0061)	0.9234(0.0038)

**Table 8:** The results of ablation experiment

Dataset	Model	AUC(std)	ACC(std)	Recall(std)	Precision(std)	AUPR(std)
DrugBank	RSGCL-DTI	0.9315(0.0065)	0.8658(0.0142)	0.8912(0.0334)	0.8683(0.0122)	0.9528(0.0047)
	RSGCL-DTI-RF	0.9213(0.0033)	0.8624(0.0015)	0.8741(0.0083)	0.8752(0.0072)	0.9323(0.0052)
	RSGCL-DTI-SF	0.9234(0.0042)	0.8526(0.0023)	0.8797(0.0014)	0.8679(0.0032)	0.9215(0.0046)
C.elegans	RSGCL-DTI	0.9946(0.0008)	0.9871(0.0026)	0.9826(0.0056)	0.9924(0.0063)	0.9969(0.0004)
	RSGCL-DTI-RF	0.9763(0.0011)	0.9127(0.0011)	0.9029(0.0546)	0.9319(0.0405)	0.9857(0.0065)
	RSGCL-DTI-SF	0.9912(0.0034)	0.9607(0.0048)	0.9634(0.0086)	0.9595(0.0024)	0.9925(0.0016)
Human	RSGCL-DTI	0.9963(0.0007)	0.9703(0.0026)	0.9715(0.0043)	0.9677(0.0033)	0.9925(0.0006)
	RSGCL-DTI-RF	0.9939(0.0003)	0.9788(0.0032)	0.9827(0.0008)	0.9738(0.0068)	0.9951(0.0006)
	RSGCL-DTI-SF	0.9905(0.0013)	0.9548(0.0050)	0.9606(0.0097)	0.9650(0.0094)	0.9908(0.0009)
BioSNAP	RSGCL-DTI	0.9605(0.0025)	0.8899(0.0041)	0.8889(0.0097)	0.8947(0.0111)	0.9572(0.0046)
	RSGCL-DTI-RF	0.8861(0.0040)	0.7885(0.0108)	0.6591(0.0293)	0.8933(0.0095)	0.8976(0.0042)
	RSGCL-DTI-SF	0.9186(0.0015)	0.8503(0.0042)	0.8588(0.0112)	0.8465(0.0056)	0.9223(0.0412)

### Parameter analysis

In this experiment, we performed parameter analysis using the DrugBank dataset to assess how the number of epochs and different node feature initialization strategies in graph contrastive learning affect model performance.

#### The analysis of the number of epochs

In graph contrastive learning, the differences between the corresponding nodes in different views are minimized to make their representations more similar. The degree of this similarity affects the accuracy of the downstream DTI prediction task. Furthermore, in graph contrastive learning this similarity is closely associated with the number of epochs. Hence, we tested the variation in predictive performance under different numbers of epochs: 1000, 2000, and 3000. As indicated by the results in Table 9, the model performance improves with more epochs, but stabilizes when the number exceeds 2000. Therefore, the number of epochs for graph contrastive learning module is set to 2000 during the experiments.

**Table 9:** The impact of the number of epochs on DTI prediction

Epoch	AUC	ACC	Recall	Precision
1000	0.9277	0.8590	0.8861	0.8641
2000	**0.9316**	0.8648	0.8838	**0.8721**
3000	0.9315	**0.8658**	**0.8912**	0.8683

#### The analysis of node feature initialization strategies

In this section, we analyze the impact of the node feature initialization strategy on predictive performance. In graph contrastive learning, the selection of initial node features is a crucial factor that influences predictive performance. It should be noted that different initial node feature matrices include different prior information, so different node feature initialization strategies may have an impact on the learning of node feature representations. Therefore, we designed two node feature initialization strategies: a uniform initialization strategy and a random initialization strategy, to study the impact of node feature initialization strategies on predictive performance. The uniform initialization strategy is to set the same random initial features for all nodes in contrastive views. This strategy considers that the relational features are subject to the topology of the relational similarity network. The difference between the node relational features derived from the same initial node features depends only on the network topology information. This strategy more accurately reflects the topological relations between nodes. The random initialization strategy uses the random function to generate different vectors of the same length for each node in the contrastive views as their initial features. In particular, the drug node is initialized by a 167-dimensional random vector, while the protein node is initialized by a 256-dimensional random vector. Compared with the uniform initialization strategy, the random initialization strategy takes into account the differences between nodes, which leads to more focus on the features of individual nodes when learning the network topology. The experiment is conducted on the DrugBank dataset, in which 2000 epochs are used for graph contrastive learning. The experimental results in Table 10 demonstrate that predictive performance using the uniform initialization strategy is significantly lower than the random initialization strategy. We argue that by assigning different initial features to each node, the model can be more flexible in capturing the differences and diversity among nodes, thus enhancing its ability to model relationships between nodes.

**Table 10:** The impact of node feature initialization strategy on DTI prediction

Initialization strategy	AUC	ACC	Recall	Precision
Uniform initialization strategy	0.8217	0.7245	0.6458	0.8342
Random initialization strategy	**0.9316**	**0.8648**	**0.8838**	**0.8721**

### Case study

In order to assess the ability of RSGCL-DTI to make predictions about unknown DTI, we followed AMGDTI [45] and utilized the DrugBank dataset as the training set. We collected 80 drug–target pairs from the online DrugBank database as positive samples. To construct the negative samples, we randomly paired these drugs with their non-interacting targets, ensuring that there were neither positive samples nor negative samples in the training set, resulting in a total of 4000 negative examples. Finally, these positive and negative samples were mixed to obtain a test set with a positive to negative ratio of 1:50. In this experiment, the AUPR of the models RSGCL-DTI, MCANet, HyperAttentionDTI, MCL-DTI, GIFDTI, MHSADTI, CoaDTI, and BINDTI are 0.5130, 0.5002, 0.4797, 0.2018, 0.5056, 0.4591, 0.1502, and 0.3855, respectively. We also analyzed the 80 drug–target pairs with the highest scores predicted by the four models with the highest AUPR metrics: RSGCL-DTI, MCANet, GIFDTI, and HyperAttentionDTI. Among the top 80 ranked drug–target pairs, RSGCL-DTI demonstrated superior predictive performance by accurately identifying 46 positive cases, outperforming MCANet and GIFDTI (both with 41 correct predictions) and HyperAttentionDTI (42 correct predictions). We also listed the top 20 drug–target pairs in Table 11.

**Table 11:** The top 20 predictions of RSGCL-DTI

Rank	Drug ID	Protein ID	Label
1	DB02659	P00326	1
2	DB13345	P07550	1
3	DB13345	P08588	1
4	DB02613	P06276	0
5	DB12267	P00533	1
6	DB04216	P17812	0
7	DB00338	P35354	0
8	DB12140	P06239	0
9	DB12695	P06753	1
10	DB12695	Q92598	1
11	DB12141	O95263	0
12	DB08231	Q16769	0
13	DB00142	O43424	1
14	DB00142	O00222	1
15	DB00142	O00341	1
16	DB00142	O14841	1
17	DB00142	O15067	1
18	DB12267	P00519	1
19	DB04794	P12931	0
20	DB02379	P02144	1

## Conclusion and future work

The prediction of DTI is an essential component of drug repurposing and the development of new drugs. In this work, we present a novel RSGCL-DTI, which employs the latent similarity relations of drugs and proteins and their chemical structure to calculate their feature representation. The RSGCL-DTI first uses known drug–protein interaction data to create a drug–protein heterogeneous graph that is transformed into relational similarity networks for drugs and proteins, and employs graph contrastive learning to obtain the relational features for drugs and target proteins, which are combined with their chemical structure features extracted by GNN and CNN, respectively to enhance the DTI predictive performance. In order to comprehensively evaluate the performance of RSGCL-DTI from multiple perspectives, eight SOTA DTI prediction models are selected as baselines, and experimental comparisons, ablation experiments and parameter analysis are conducted. The experimental results show that RSGCL-DTI has a significant advantage over the baselines, demonstrating that the combination of structural and relational features can effectively improve DTI prediction performance.

Our proposed model consists of two key modules. One of them is the graph contrastive learning module, designed to extract relational features. In the future, we plan to leverage large language models to compute feature representations of drugs and proteins, and subsequently construct feature similarity networks based on these pre-trained features. We will then apply contrastive learning to these networks to refine and enhance their feature representations, ultimately improving the performance of DTI prediction.

**Key points**

- In this paper, we present RSGCL-DTI.
- To model similarity relationship among proteins and among drugs, we constructed two relational similarity networks based on the known DTI networks and used contrastive learning to capture the relational features of proteins and drugs. Meanwhile, we extracted the relational features of proteins and drugs using CNN and D-MPNN, respectively.
- Experiments on several balanced and imbalanced datasets show that RSGCL-DTI outperforms several existing SOTA models in terms of prediction performance and has excellent generalization capabilities.

## Data Availability

Codes and datasets of RSGCL-DTI are available at https://github.com/tangjlh/RSGCL-DTI.
